# Conversion of autoimmune hypothyroidism to hyperthyroidism

**DOI:** 10.1186/1756-0500-7-489

**Published:** 2014-08-03

**Authors:** Saira Furqan, Naeem-ul Haque, Najmul Islam

**Affiliations:** 1 Section of Endocrinology, Diabetes & Metabolism, Department of Medicine, Aga Khan University Hospital, Stadium Road, P.O. Box 3500, Karachi, Pakistan

**Keywords:** Hypothyroidism, Hyperthyroidism, Over-replacement, Conversion

## Abstract

**Background:**

Graves’ disease and Hashimoto’s thyroiditis are the two autoimmune spectrum of thyroid disease. Cases of conversion from hyperthyroidism to hypothyroidism have been reported but conversion from hypothyroidism to hyperthyroidism is very rare. Although such cases have been reported rarely in the past we are now seeing such conversions from hypothyroidism to hyperthyroidism more frequently in clinical practice.

**Case presentation:**

We are reporting three cases of middle aged Asian females who presented with classical symptoms of hypothyroidism and the investigations showed elevated thyroid stimulating hormone with positive thyroid antibodies. Diagnosis of autoimmune hypothyroidism was made and thyroxine replacement therapy was initiated. Patients became asymptomatic with normalization of thyroid stimulating hormone level. After few years they developed symptoms of hyperthyroidism with suppressed thyroid stimulating hormone level. Over replacement of thyroxine was considered and the dose of thyroxine was decreased, but they remain symptomatic. After gradual decrease in the dose of thyroxine it was stopped finally. Even after few months of stopping thyroxine, the symptoms of hyperthyroidism did not improve and the biochemical and imaging modalities confirmed that the patients have developed hyperthyroidism. Anti-thyroid treatment was then started and the patients became symptom free.

**Conclusion:**

High index of suspicion should be there for possible conversion of hypothyroidism to hyperthyroidism if a patient with primary hypothyroidism develops persistent symptoms of hyperthyroidism. Otherwise it can be missed easily considering it as an over replacement with thyroid hormone.

## Background

Autoimmune thyroid disease is one of the commonest autoimmune diseases, affecting 2-4% of women and 1% of men [1-3]. Grave’s disease and Hashimoto’s thyroiditis are the two autoimmune spectrum of thyroid disease. They have a complex etiology with poorly understood pathogenesis. The pathogenesis is influenced by certain environmental, hormonal and genetic factors. In both autoimmune hypothyroidism and Grave’s disease, genetic factors play a major role [4]. Cases of conversion from hyperthyroidism to hypothyroidism have been reported [5] but conversion from hypothyroidism to hyperthyroidism is thought to be very rare, although reported [6]. We are reporting three cases of autoimmune hypothyroidism that have converted to hyperthyroidism requiring anti-thyroid treatment.

## Cases presentation

### Case 1

A 36 years old female presented with a 3 months history of easy fatigability, cold intolerance, polymenorrhagia, constipation and weight gain in the beginning of year 2005. On examination she had bradycardia and dry skin. The thyroid gland was palpable, non-tender mostly diffuse but some nodular feeling at upper pole of left lobe. Clinical suspicion of primary hypothyroidism was made and it was confirmed by TSH value of greater than 50 uIU/ml with FT4 of less than 0.30 ng/dl and positive thyroid antibodies. Thyroxine was started at a dose of 100 mcg/day. Gradually the requirement of thyroxine decreased and by the end of 2005 onwards she maintained her TSH within normal range on 50 mcg/day of thyroxine. In the beginning of 2008 the dose was further reduced to 25 mcg/day but again towards the end of 2009 thyroxine dose was increased to 50 mcg/day because of slightly increased TSH of 8.86 uIU/ml. Slightly more than a year later in the beginning of 2011, she presented with weight loss of 3 kg with a feeling of anxiety and associated tremors of hands. TSH at this time was less than 0.005 uIU/ml with a FT4 of 2.4 ng/dl, confirming the state of thyrotoxicosis. Thyroxine was stopped and patient was observed intermittently over a period of 6 months. She remained clinically and biochemically hyperthyroid with a repeat TSH of <0.005 uIU/ml and an FT4 of 2.66 ng/dl. Thyroid scintigraphy with technetium 99 was done and it showed an increased homogenous tracer uptake. Finally she was started on Neomercazole in mid of 2011and remains on it till to date.

### Case 2

46 years old female, mother of 3 children diagnosed as having primary hypothyroidism on the basis of clinical symptoms of easy fatigability, weight gain and increase sleep and a serum TSH level of >75 uIU/ml, FT4: 0.25 ng/dl and strongly positive thyroid antibodies in December 2002. Thyroxine 50 mcg/day was started which she continued. After 2 years she presented to us with complain of weight loss. Her serum TSH level was 0.035 uIU/ml and a T4: 7.84 ng/dl so thyroxine was stopped. She came for follow up after 5 months with serum TSH: 0.010 uIU/ml off thyroxine. After another 6 months her serum TSH remained suppressed with a value of 0.018 uIU/ml and an FT4 of 1.18 ng/dl and she remained off thyroxine. After further 2 years her TSH was 0.837 uIU/ml, FT4 0.922 ng/dl and FT3 3.06. And after 1 year she presented with a TSH level of <0.005 uIU/ml, raised FT4 and positive thyroid microsomal antibodies (1: 1600). Techniteum 99 thyroid scan showed diffusely increased homogenous tracer uptake. Neomercazole was started at the dose of 5 mg/day which was gradually increased to 10 mg/day. She followed up after 4 months with TSH: 0.01 uIU/ml, T4: 5.84 ng/dl, T3: 1.99 ng/dl. Neomercazole was continued. Then she came for follow up after 1 year, at this time she was clinically euthyroid but biochemically hypothyroid with serum TSH: 14.89 uIU/ml, FT4:0.65 ng/dl. Neomercazole was stopped and advised to repeat TFT’s after 8 weeks. She came after 4 months with TSH: 3.98 uIU/ml, so no treatment was advised. After a period of 1 year she again came with complains of weight loss and palpitations. At this time her TSH: < 0.005 uIU/ml, FT4: 2.25 ng/dl. Neomercazole was restarted and Radioactive iodine 131 ablation planned.

### Case 3

A 43 year old obese female, married, mother of 4 children was diagnosed as having hypothyroidism in the beginning of year 2011 when she felt lethargic and her TSH level was raised to 72 uIU/ml. She was started on thyroxine 150 mcg/day which was later on decreased to 100 mcg when her TSH level was suppressed to 0.5 uIU/ml after 1 month. After further 4 weeks her serum TSH came out to be further suppressed at <0.005 uIU/ml so thyroxine was stopped. The patient came for follow up after 8 weeks with serum TSH of 0.2 uIU/ml and T4 of 16.20 ng/dl (4.5-11.3). She then lost to follow up and returned in September 2011 with Thyroid function test showing serum TSH of <0.005 uIU/ml and FT4: 2.36 ng/dl. Thyroid antibodies were strongly positive. At this time she also had complained of palpitations, increased appetite, increased bowel movements, decreased sleep and tremors of hands. On examination she had no goiter and eye signs but had fine tremors of hands. She was advised to start carbimazole.

## Discussion

In this case series, we have discussed 3 patients who developed hyperthyroidism after few months or years of established hypothyroidism. Initially they were proven to have autoimmune hypothyroidism on the basis of clinical and biochemical parameters, with multiple elevated levels of serum TSH and strongly positive thyroid antibodies. They remained euthyroid on thyroxine therapy for quite a while. Afterwards they developed clinical and biochemical hyperthyroidism, for which thyroxine was gradually decreased and finally stopped, but they remained hyperthyroid even after months of stopping thyroxine. Finally they needed anti-thyroid treatment.

Primary Hypothyroidism once established has been considered as a stable or gradually progressing disease for which lifelong replacement therapy is needed but very occasionally the condition might reverse. Review of literature showed that so far 37 cases has been reported where hypothyroidism later on converted to hyperthyroidism. It was first reported in 1959 by Joplin and Fraser [7]. In 1960 Doniach et al. [8] reported the same. Gavras and Thomson [9] documented two cases that developed hyperthyroidism while they were taking thyroxine therapy for hypothyroidism. Others have also reported the same [10-32]. Takasu et al. [6] published a case series of 8 patients who were initially hypothyroid but later on converted to hyperthyroidism. Bando et al. and Ruchala et al. reports cases of hyperthyroidism following hypothyroidism in cases of thyroid hemiagenesis [33,34].

Why a patient with established hypothyroidism later on converts to hyperthyroidism is not well understood. Several mechanisms have been postulated behind this conversion. One important mechanism hypothesized is the presence of different autoantibodies that includes thyroid stimulating antibodies, thyroid stimulation blocking antibodies and the response of thyroid gland to these antibodies [35].

The transition from TSH blocking antibodies to thyroid stimulation antibodies may be the cause of hyperthyroidism. Some patients actually have both antibodies specific to Hashimoto’s and Graves’ disease, which puts the thyroid into a push-pull situation, where it cycles up and down through hypothyroidism and hyperthyroidism. Other possible explanation for this transition from hypothyroidism to hyperthyroidism is that the auto-immune tissue damage, initially severe enough to cause thyroid hypofunction, recovered sufficiently to allow subsequent stimulation by TSAb [36]. Unfortunately these antibody testing haves not been done in our patients due to non-availability of these tests at our set up.

Although there are anecdotal reports that showed that patients diagnosed with hypothyroidism and taking treatment for it, developed hyperthyroidism later on. It was thought to be a rare phenomenon but we have identified 3 patients in a short period of time so it seems that it is becoming a common occurrence and no more a rare problem. So the index of suspicion should be high.

## Conclusion

These cases demonstrate that diagnosis of primary hypothyroidism does not necessarily means lifelong replacement of thyroid hormone.

High index of suspicion should be there for a possible conversion of hypothyroidism to hyperthyroidism if a patient with primary hypothyroidism develops persistent symptoms of hyperthyroidism. Otherwise it can be missed easily considering it as an over replacement with thyroid hormone.

## Consent

Written informed consent was obtained from the patients for publication of this Case report and accompanying image. A copy of the written consent is available for review by the Editor of this journal.

## Abbreviations

TSH: Thyroid stimulating hormone; T4: Total Thyroxine; T3: Tri-iodothyronine; FT4: Free thyroxine level; TSAb: Thyroid stimulating antibodies.

## Competing interests

We, the authors of this case series declare that there is no conflict of interest that could be perceived as prejudicing the impartiality of the research reported. We also declare that there is no financial or other potential conflict of interest.

## Authors’ contributions

SF drafted the manuscript and searched the literature. N-l-H was actively involved in the patients’s management and revised the manuscript. NI was primarily responsible for the conception and revision and editing of the manuscript. All the authors read and approved the final manuscript.

## Funding

As it is a case series, it did not receive any specific grant from any funding agency in the public, commercial or not-for-profit sector.
